# Utility of antioxidants during assisted reproductive techniques: an evidence based review

**DOI:** 10.1186/1477-7827-12-112

**Published:** 2014-11-24

**Authors:** Ashok Agarwal, Damayanthi Durairajanayagam, Stefan S du Plessis

**Affiliations:** Center for Reproductive Medicine, Glickman Urological & Kidney Institute, Cleveland Clinic, Cleveland, OH 44195 USA; Discipline of Physiology, Faculty of Medicine, MARA University of Technology, Sungai Buloh, Selangor 47000 Malaysia; Division of Medical Physiology, Faculty of Medicine and Health Sciences, Stellenbosch University, Tygerberg, 7505 South Africa

**Keywords:** Reactive oxygen species, Oxidative stress, Antioxidants, Assisted reproductive technology, In vitro fertilization, Intracytoplasmic sperm injection, ART outcome

## Abstract

Assisted reproductive technology (ART) is a common treatment of choice for many couples facing infertility issues, be it due to male or female factor, or idiopathic. Employment of ART techniques, however, come with its own challenges as the *in vitro* environment is not nearly as ideal as the *in vivo* environment, where reactive oxygen species (ROS) build-up leading to oxidative stress is kept in check by the endogenous antioxidants system. While physiological amounts of ROS are necessary for normal reproductive function *in vivo*, *in vitro* manipulation of gametes and embryos exposes these cells to excessive ROS production either by endogenous or exogenous environmental factors. In this review, we discuss the sources of ROS in an *in vitro* clinical setting and the influence of oxidative stress on gamete/embryo quality and the outcome of IVF/ICSI. Sources of ROS and different strategies of overcoming the excessive generation of ROS *in vitro* are also highlighted. Endogenously, the gametes and the developing embryo become sources of ROS. Multiple exogenous factors act as potential sources of ROS, including exposure to visible light, composition of culture media, pH and temperature, oxygen concentration, centrifugation during spermatozoa preparation, ART technique involving handling of gamete/embryo and cryopreservation technique (freeze/thawing process). Finally, the use of antioxidants as agents to minimize ROS generation in the *in vitro* environment and as oral therapy is highlighted. Both enzymatic and non-enzymatic antioxidants are discussed and the outcome of studies using these antioxidants as oral therapy in the male or female or its use *in vitro* in media is presented. While results of studies using certain antioxidant agents are promising, the current body of evidence as a whole suggests the need for further well-designed and larger scale randomized controlled studies, as well as research to minimize oxidative stress conditions in the clinical ART setting.

## Background

Infertility, a disease of the reproductive system defined by the failure to achieve a clinical pregnancy after 12 months or more of regular unprotected sexual intercourse [1], affects 15% of all couples, with nearly a quarter of cases being without an identifiable causative factor [2]. Medical treatment for infertility include *in vitro* fertilization (IVF) and intracytoplasmic sperm injection (ICSI), which are the two most common interventions used in assisted reproductive technology (ART) [3].

Successful ART outcome, including fertilization and clinical pregnancy resulting in live birth, is influenced by a multitude of factors - among which reactive oxygen species (ROS) plays a significant role [4]. The consequent development of oxidative stress is among the chief causes of defective gametes or poorly-developing embryos in ART [5]. This occurs because the IVF process performed in a clinical laboratory setting cannot recreate the exact conditions under which natural fertilization takes place [6]. Among the crucial factors lacking in assisted reproduction procedures is the tight control of ROS levels maintained within physiological concentration by antioxidants *in vivo* [3].

In order to optimize gamete/embryo quality and improve ART outcome, deliberate preventive measures are necessary to reduce any incidental build-up of ROS leading to oxidative stress development during ART. One method to achieve this would be by enhancing the antioxidant capacity of the gamete and embryo against the harmful assault of oxidation. In this paper, we will review the sources of oxidative stress and the use of antioxidants in a clinical ART setting to minimize the detrimental effects of oxidative stress on gamete and/or embryo during assisted reproduction.

## Free radicals, reactive oxygen species and oxidative stress

Free radicals are molecules or atoms with an odd or unpaired number of valence electrons. Although necessary for physiological bodily functions, free radicals are harmful in larger amounts and are involved in the pathophysiology of various diseases [6]. Free radicals are extremely reactive and participate in chain reactions that cause other molecules to become unstable, which generate even more free radicals [5].

ROS comprise both free radical and non-free radical oxygen-derived reactive molecules. ROS are constantly generated, as part of normal aerobic life, during the intermediate steps of oxygen reduction along the mitochondrial electron transport chain [7]. Formation of ROS also occurs as necessary intermediates during various enzymatic reactions. Common forms of ROS include superoxide anion radical (O_2_^•-^), hydroxyl radical (^•^OH), hydrogen peroxide (H_2_O_2_) and singlet oxygen (^1^O_2_). Reactive nitrogen species (a subset of ROS) include nitric oxide (NO^•^) and the peroxynitrite anion (ONOO^−^) [8]. ROS in high concentrations cause cellular toxicity and can impair the spermatozoon’s ability to fertilize the oocyte [9]. However, small amounts of ROS are required for the regulation of various gamete functions [10, 11].

Oxidative stress develops upon an imbalance between systemic production of ROS and the ability to either readily detoxify ROS (antioxidant defenses) or repair the ensuing damage resulting from lipid peroxidation, DNA damage and apoptosis [12]. Prevention of oxidative stress is vital in order to maintain normal reproductive function [13]. Both male and female reproductive systems possess antioxidant defense mechanisms that facilitate the quenching of ROS, and maintain equilibrium between pro- and anti-oxidants. This confers protection from oxidative damage to the gonadal cells and gametes, which is vital in the upkeep of normal reproductive function.

## Oxidative stress in the ART setting

Despite the advancement of ART techniques, gametes and embryos when handled, prepared and manipulated for ART procedures, are exposed to various potential ROS-inducing factors. *In vitro*, the risk of oxidative stress development is greater than *in vivo* [13] and its negative impact may be amplified due to the lack of physiological defense mechanisms, absence of natural antioxidants and the presence of multiple potential sources of ROS [4]. These sources of ROS during ART procedures could either be endogenously from gametes or via exogenous environmental factors [5]. However, unless measures are taken to curb ROS production, both the endogenous and exogenous sources of ROS will ultimately lead to the development of oxidative stress, which would then negatively impact on fertilization rates and pregnancy outcome.

### Sources of ROS

#### In vivo

Spermatozoa, oocytes and embryos rely on mitochondrial oxidative phosphorylation for energy, a process which is subsequently accompanied by ROS generation [5].

##### Originating from the male

Normal human spermatozoa function such as maturation, capacitation, hyperactivation, acrosome reaction and oocyte fusion are facilitated by physiological levels of ROS [14]. However, excess ROS is detrimental to the spermatozoa functionality and could lead to male infertility [15]. In human semen, immature spermatozoa and leukocytes are the two main endogenous potential sources of ROS [16]. Spermatozoa are particularly susceptible to oxidative stress as its cell membranes are rich in polyunsaturated fatty acids, making it more vulnerable to oxygen-induced damage and lipid peroxidation. Furthermore, mature spermatozoa lack cytoplasmic enzymes and antioxidant defense mechanisms [17].

##### Immature spermatozoa

During spermatogenesis damaged spermatozoa undergo arrested spermiogenesis. This causes them to maintain excess residual cytoplasm, which can activate the NADPH system. As such, spermatozoa with cytoplasmic droplets act as a potential contributor to ROS production [18]. Immature spermatozoa with excess cytoplasm around its midpiece are functionally defective having impaired motility and abnormal morphology, which impacts negatively on its fertilization potential [19].

##### Leukocytes

Leukocytes are the predominant source of ROS during spermatozoa preparation, as they are able to produce up to 1000 times more ROS than spermatozoa in human semen [20, 21]. Originating from the prostate gland and seminal vesicles, peroxidase-positive leukocytes include polymorphonuclear leukocytes and macrophages [22]. During infection or inflammation processes *in vivo*, leukocytes release large amounts of superoxide when conquering pathogens [4]. Seminal leukocytes also stimulate spermatozoa to produce ROS [3].

##### Varicocele

Varicocele is the abnormal dilatation of veins in the pampiniform plexus surrounding the spermatic cord. It is believed to be the most common and treatable cause of male factor infertility, however a recent Cochrane review suggests that these assumptions are inconclusive as the quality of the available evidence is very low [23]. Varicocele of a higher grade is associated with greater amounts of seminal ROS [24]. Infertile men with varicocele have increased oxidative stress levels and lowered antioxidant concentrations [25].

##### Originating from the female

Physiological levels of ROS are likely to play a role in several aspects of female reproduction including ovarian steroidogenesis, oocyte maturation, folliculogenesis, ovulation and luteolysis [26, 27].

##### Oocyte

Oocyte quality is correlated with 8-hydroxy-2’-deoxyguanosine (8-OHdG) (a biomarker of oxidative stress induced-DNA damage) levels in granulosa cells [28]. Poor oocyte quality would lead to compromised embryo development. In the oocyte, ROS levels when present in excess, can disrupt the oocyte cytoskeleton, alter microtubule function, cause chromosomal scattering and aneuploidy [5]. These effects could negatively impair ART outcome.

##### Cumulus mass cells

Cumulus cells originate from relatively undifferentiated granulosa cells. The cumulus oophorus encircles the oocyte and is made up of the cumulus cells and extracellular matrix [29]. Cumulus cells closely interact and provide support to the developing and maturing oocyte, shares the oocyte’s microenvironment and minimize damage by ROS. Cumulus cells are able to produce antioxidants, such as superoxide dismutase (SOD), which are suggested to protect the oocyte from ROS-induced damage [30]. Higher SOD levels in cumulus cells are associated with ART outcome success [31]. Increased levels of 8-OHdG (an oxidative stress by-product) in cumulus cells yielded lower oocyte fertilization rates and poorer embryo quality [28].

##### Follicular fluid

Follicular fluid is secreted by the follicular theca and granulosa cells, and fills the antral follicle. Low levels of follicular fluid ROS may be used to predict potential success of IVF [32, 33]. Pregnancy outcome after ICSI is negatively associated with high levels of follicular fluid ROS, but is positively associated with follicular fluid total antioxidant capacity [34]. The ROS upper reference limit above which viable embryo formation became unsatisfactory was calculated to be around 107 cps/400 μl follicular fluid in women with tubal factor infertility, endometriosis and polycystic ovarian syndrome (PCOS). Not only was fertilization rate and percentage of grades I and II embryo formation higher in those patients with follicular fluid ROS levels less than the set limit, when compared to those above the limit, but DNA fragmented embryos were also significantly lower [35]. As such, the ROS level in follicular fluid seems to provide an indication of embryo formation and quality [36].

##### Originating from the embryo

The fast developing embryo produces energy via ATP generation through mitochondrial oxidative phosphorylation and glycolysis. As it develops, the embryo is capable of producing ROS through several pathways, namely oxidative phosphorylation, NADPH and xanthine oxidase systems [37]. ROS generation is particularly excessive during embryonic genome activation, embryonic compaction and hatching as these processes demand greater amounts of energy [38]. Conversely, embryos exposed to high ROS levels are of lower quality and run the risk of early embryonic developmental block and retardation [39]. Although ROS production was reportedly greater in embryos cultured *in vitro* compared to those developing *in vivo* [40], it remains uncertain if and how much the ART procedure itself (techniques and conditions employed) contributed to the higher levels of oxidative stress [5].

#### In vitro

**Figure 1:**
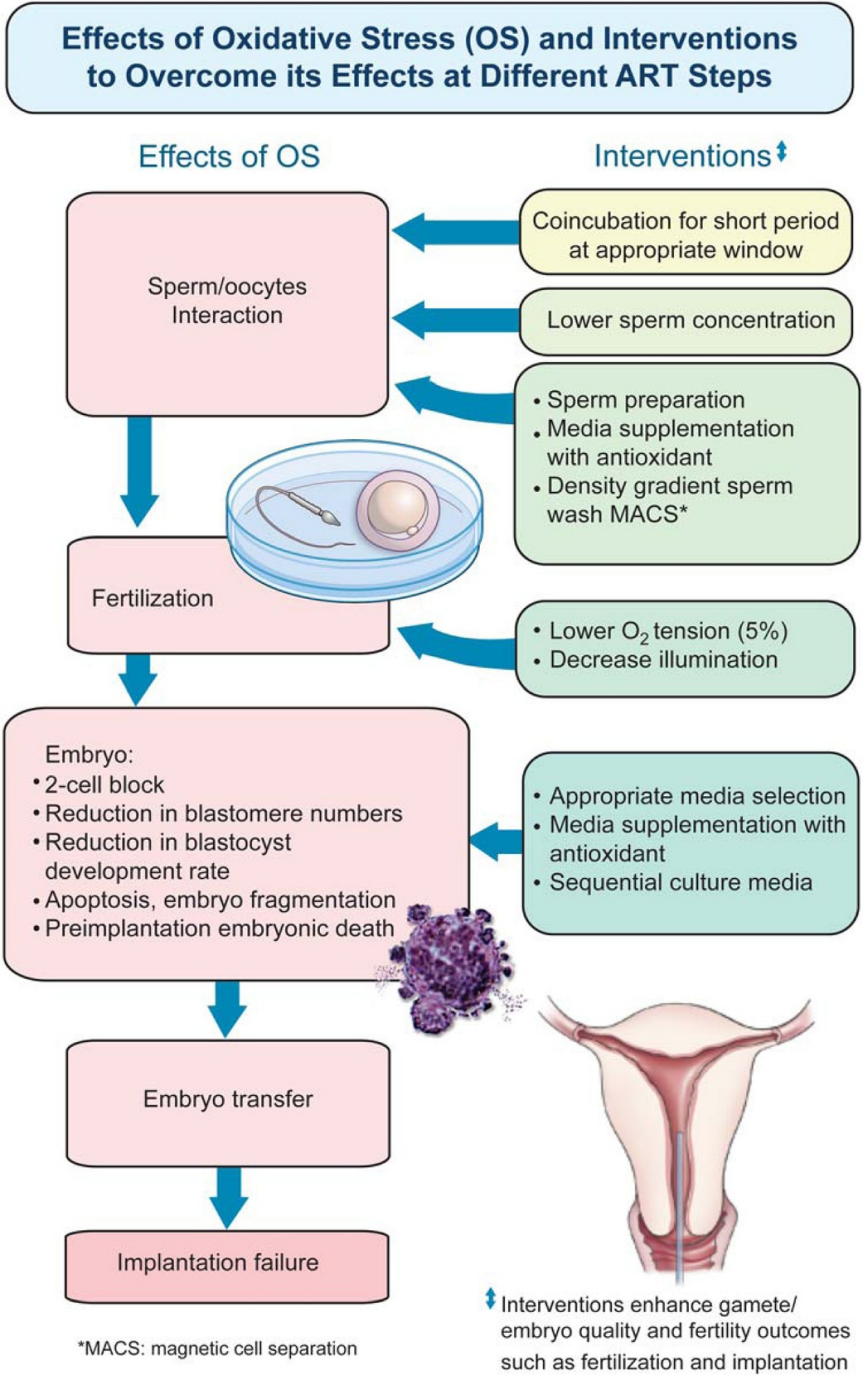
**Potential sources of oxidative stress** ***in vitro*** **in a typical clinical ART setting.** In a typical ART setting, the potential sources of oxidative stress *in vitro* include endogenous and exogenous (external/environmental factors). The gametes and pre-implantation embryo have the potential to generate ROS (endogenous sources). Exogenous factors such as visible light; centrifugation, cryopreservation (freeze/thawing), culture media; oxygen concentration, pH and temperature; and the *in vitro* fertilization-embryo transfer technique/process itself contributes to ROS production during ART.

Figure 1 depicts the potential sources of ROS and oxidative stress *in vitro* in a typical clinical ART setting.

##### Visible light

*In vitro* handling of gametes and embryos involves the inevitable exposure to visible light (400–700 nm), from both microscope and from ambient lighting (laboratory/fluorescent light and daylight/indirect sunlight) [41, 42]. Light within the visible spectrum (visible light) has detrimental effects on gametes and developing embryo. The negative impact of visible light is influenced by duration of exposure, intensity and spectral composition of light [42].

Blue light (400-500 nm) is particularly more damaging than visible light with longer wavelengths, as blue light could generate hydrogen peroxide and alter enzymes in the respiratory chain [43, 44]. Mouse embryo exposed to blue light had reduced blastocyst formation rates, higher blastomeric apoptosis rates and higher ROS production in morula [45]. The use of light filters on inspection microscopes (which cuts off light <500 nm) [42, 46], illumination levels kept at a minimum without compromising visual inspection and shorter inspection time could help curb these effects [42].

Light exposure is measured as units of illumination intensity (lux) or by the level of irradiation (W/m^2^). Using low illumination levels (100 lux from microscope, 20 lux from ceiling) during human embryo manipulation throughout *in vitro* fertilization-embryo transfer (IVF-ET) procedures (and other measures to minimize oxidative stress development *in vitro*) in 110 IVF cycles yielded a relatively high blastulation rate [46].

In a recent *in vitro* study, porcine parthenogenetically-activated embryos that were developed in culture medium with prior 24 h-exposure to ambient light yielded a higher percentage of blastocysts with poor morphology [41]. Further, activated-oocytes that had prior 1 h-exposure to ambient light formed fewer and lower quality blastocysts. This detrimental effect of light exposure on blastocysts was found to increase with time [41]. Several other studies have documented the negative impact of visible light exposure on animal embryo development [43, 47–50].

Light irradiation (40 mW/cm^2^ visible light or 400 to 800 nm with maximum energy at 600 nm for 3 minutes) of human spermatozoa in capacitation media increased hyper-activated motility, without enhancing total motility [51]. As hyper-activated motility escalates the swimming speed of spermatozoa and helps produce adequate penetration force [52], development of hyper-activated motility during the spermatozoa capacitation process may be critical to ensure successful fertilization [53]. However, production of ROS in these spermatozoa increased upon 1 to 3 minutes of light exposure [51].

##### Culture media

The composition of media used during the culture of human oocytes and pre-implantation embryos has a direct influence on embryo quality and subsequently ART success [11]. Presence of metallic ions (iron, Fe^2+^ and copper, Cu^2+^) in culture media could trigger ROS-generating reactions within the cells [37], and the rate of ROS formation varies with the composition of culture media [35]. Addition of metal chelators (e.g. EDTA) may reduce ROS formation [54, 55], however, additional supplements (e.g. albumin) may instead cause a build-up of oxygen load [5]. Supplementation of culture media with antioxidants (e.g. ascorbic acid, alpha-tocopherol) could help alleviate the adverse effects of ROS on gametes [56]. Key findings of studies using antioxidants *in vitro* in media are summarized in Table 1.

**Table 1:** **Study outcomes involving** ***in vitro*** **supplementation of various antioxidants during ART protocol**

Antioxidant	Study type	Patient population	Intervention (therapeutic approach)	Control group (daily dose)	Outcome/effect of intervention/effect on parameters	Reference
*Vitamin E*	Prospective	Sperm from normozoospermic and asthenozoospermic men	5 mM vitamin E added to cryoprotective media prior to freeze-thaw procedure		1. Improvement in post-thaw motility	Kalthur et al. [57]
					2. Improvement in DNA integrity	
*Vitamin E*	Prospective	Sperm from normozoospermic men and men with abnormal sperm parameters	100 μmol or 200 μmol vitamin E added to cryopreservation media		Improved post-thaw motility of cryopreserved sperm from men with both normal and abnormal sperm parameters	Taylor et al. [58]
*Vitamin E (alpha-tocopherol)*	Prospective	Sperm from teratozoospermic men (n = 15)	Sperm prepared by swim up incubated with 40 μmol alpha-tocopherol added to media x 1 hour		1. Improved sperm motility	Keshtgar et al. [59]
					2. Increased sperm viability	
*Vitamin C*	Prospective	Sperm from male volunteers with teratozoospermia (n = 15)	Sperm prepared by swim up incubated with 600 μmol vitamin C added to media x 1 hour		1. Reduced MDA levels	Fanaei et al. 2014 [60]
					2. Reduced DNA damage	
					3. Improved sperm progressive motility	
					4. Improved sperm viability	
*Vitamin C*	Prospective	DNA damaged sperm from infertile men	10 mM ascorbic acid added to semen sample prior to adding cryomedia	Unsupplemented cryomedium	1. No change in post-thaw sperm concentration or morphology	Branco et al. [61]
					2. Reduced number of sperm with cryopreservation-induced DNA damage in infertile men	
*Vitamin C*	Prospective	Sperm from patients undergoing semen analysis (n = 134)	Supplementation of cryomedium with ascorbate or 100 μmol/L AA2G (ascorbic acid-2-glucoside) (stabilized form of ascorbate)	Unsupplemented cryomedium	Improved post-thaw sperm motility	Jenkins et al. [62]
*Coenzyme Q*_*10*_	Prospective	Sperm from asthenozoospermic men (n = 22)	HAM’s medium alone, HAM’s medium +1% DMSO, HAM’s medium +5 μM CoQ10 or 50 μM CoQ10 x 24 hours	Samples with normal motility sperm (n = 16)	50 μM CoQ10 increased sperm motility of asthenozoopsermic men *in vitro*	Lewin & Lavon [63]
*Melatonin*	Experimental	Sperm from both healthy and infertile men (n = 12)	Sperm co-incubated with 1 mM melatonin x 30 minutes	No treatment	1. Increased percentage of motile and progressively motile cells	Ortiz et al. [64]
					2. Increased sperm vitality and sperm with normal morphology	
*Melatonin*	Experimental	Sperm from healthy men (n = 12)	Sperm co-incubated with 2 mM melatonin x 120 minutes	No treatment	1. Higher percentage of motility and progressive motility	du Plessis et al. [65]
					2. Increased sperm viability	
*L-Carnitine*	Experimental	Peritoneal fluid from women with endometriosis	Frozen metaphase II mouse oocytes and embryos in peritoneal fluid (from endometriosis patients) incubated with 0.6 mg/mL L-Carnitine	Peritoneal fluid (from endometriosis patients) only, peritoneal fluid (from tubal ligation patients as control) only, human tubal fluid only, L-carnitine only	1. Improved microtubule and chromosome structure in oocyte	Mansour et al. [66]
					2. Decreased level of embryo apoptosis	
*L-Carnitine*	Experimental	Embryo	0.3 mg/mL or 0.6 mg/mL L-Carnitine	Embryo culture medium without supplementation	1. Improved percentage of blastocyst development rate with 0.3 mg/mL L-carnitine	Abdelrazik et al. [67]
					2. Both 0.3 mg/mL and 0.6 mg/mL L-carnitine reduced the blocking effect of actinomycin-D, hydrogen peroxide or tumor necrosis factor alpha and reduced the level of DNA damage	

##### pH and temperature

Intracellular homeostasis is highly susceptible to changes in pH (most occur within a pH of 6 to 8), especially key processes such as protein synthesis, mitochondrial function, cytoskeletal regulation and cellular metabolism [68]. Fluctuations of hydrogen ion concentration (pH) in culture media could negatively impact spermatozoa motility, oocyte maturation and embryo development [68, 69]. Thus, to maintain the pH of culture media, incubator CO_2_ levels should be kept stable, as low CO_2_ levels tend to increase the pH of culture media [68]. Increases in pH could subject cells to oxidative stress conditions.

The use of buffers in media helps with pH maintenance, such as sodium bicarbonate during IVF procedures, and HEPES buffer for storage and handling of spermatozoa [68]. In case of room temperature-procedures such as collection of gamete, cryopreservation, ICSI and embryo transfer, external pH outside of the incubator is maintained by including another pH buffer along with handling media containing lower bicarbonate levels [68].

Incubator temperature should also be constantly maintained at human body temperature [70], as increasing temperatures decrease pH and pK_a_ levels [71], disrupt intracellular processes and may further cause ROS-induced cellular damage [72].

##### Oxygen concentration

During IVF and ICSI, pre-implantation embryos are cultured in the ART laboratory, commonly under the oxygen concentration of either atmospheric (~20%) or low (~5%) oxygen concentrations *in vitro* [73]. Compared to atmospheric (~20%) oxygen concentrations, embryo culture in lower (~5%) oxygen concentrations closer resembled physiological oxygen concentration in the oviduct and uterus (~2% to 8%) [73]. Hyperoxic conditions could enhance the activity of oxygen-dependent oxidase enzymes [37]. Thus, oxygen concentrations at atmospheric levels could generate ROS and cause the development of oxidative stress [74], thus negatively impacting embryo quality.

A Cochrane systematic review (7 studies, 2422 participants) and meta-analysis (4 studies, 1382 participants) reported that embryos developed better and were of higher quality when cultured in low (5%) oxygen concentrations, leading to improved ongoing and clinical pregnancy rates, and live birth rates. Thus, embryo culture in low (~5%) oxygen concentrations improves IVF/ICSI success rates and results in the birth of healthier babies [75]. Even among poor responders of IVF and ICSI cycles, embryos developed at low (5%) oxygen concentrations resulted in higher pregnancy rates [76, 77].

In an earlier meta-analysis of 7 randomized controlled trials (RCTs) comparing the effects of oocyte/embryo culture at low (~5%) and atmospheric (~20%) oxygen concentrations, embryos transferred on days 2 or 3 had similar implantation rates while embryos transferred on days 5 or 6 (blastocyst stage) had significantly higher implantation rates when cultured in 5% oxygen concentration. However, in this study, ongoing pregnancy rates were similar regardless of the oxygen concentration (~5% or ~20%) or day of transfer (days 2/3 or days 5/6) [78].

##### Centrifugation

In ART, centrifugation is a routine step used in spermatozoa preparation techniques to remove seminal plasma, which is a potential source of ROS [79]. However, the centrifugation process itself contributes to ROS levels, with the length of centrifugation time having a greater influence in inducing-ROS formation compared to the g-force applied [79]. Despite the initial spermatozoa quality, longer time of centrifugation exposes spermatozoa to higher temperature and causes greater detriment to sperm parameters [80]. Thus, during spermatozoa preparation protocols, addition of antioxidants such as pentoxifylline [81] in advance of the centrifugation step, could reduce centrifugation-induced ROS production and damage to processed spermatozoa [82].

##### ART technique

In the clinical setting, gamete and embryo manipulation *in vitro* during ART is a potential source of ROS production [4]. An IVF procedure involves long incubation time of spermatozoa, oocyte and its cumulus cells (which are potential ROS generators) in the fertilization medium. Conversely, ICSI has a shorter incubation period which involves only one spermatozoon and an oocyte that has been stripped of its cumulus cells. Hence, ICSI carries a lower risk of ROS production during fertilization compared to IVF [6]. However, ICSI carries a greater risk of exposing oocyte DNA to ROS-induced damage as there is a risk of transferring a small quantity of ROS-containing medium along with the spermatozoon into the oocyte [83]. Despite the selected spermatozoon having a morphologically-normal appearance, it may carry a greater risk of having DNA damage as natural spermatozoa selection are bypassed during the ICSI procedure and specifically more so as this is usually the technique of choice when spermatozoa quality is poor [84].

##### Cryopreservation (freeze/thawing)

Cryopreservation involves the preservation of gametes/embryos and whole ovarian or testicular tissues by cooling to sub-zero temperatures followed by thawing for use in ART treatments [85]. Although the use of cryoprotectants and optimized protocols seem to enhance cell viability, the freeze-thaw process is an extreme stressor that can modify the structure and integrity of the cell, e.g. spermatozoa plasma membrane [86]. During cryopreservation, freeze-thaw procedures increase DNA oxidative damage and fragmentation levels, causing post-thaw spermatozoa to have poorer motility and viability [87, 88].

**Figure 2:**
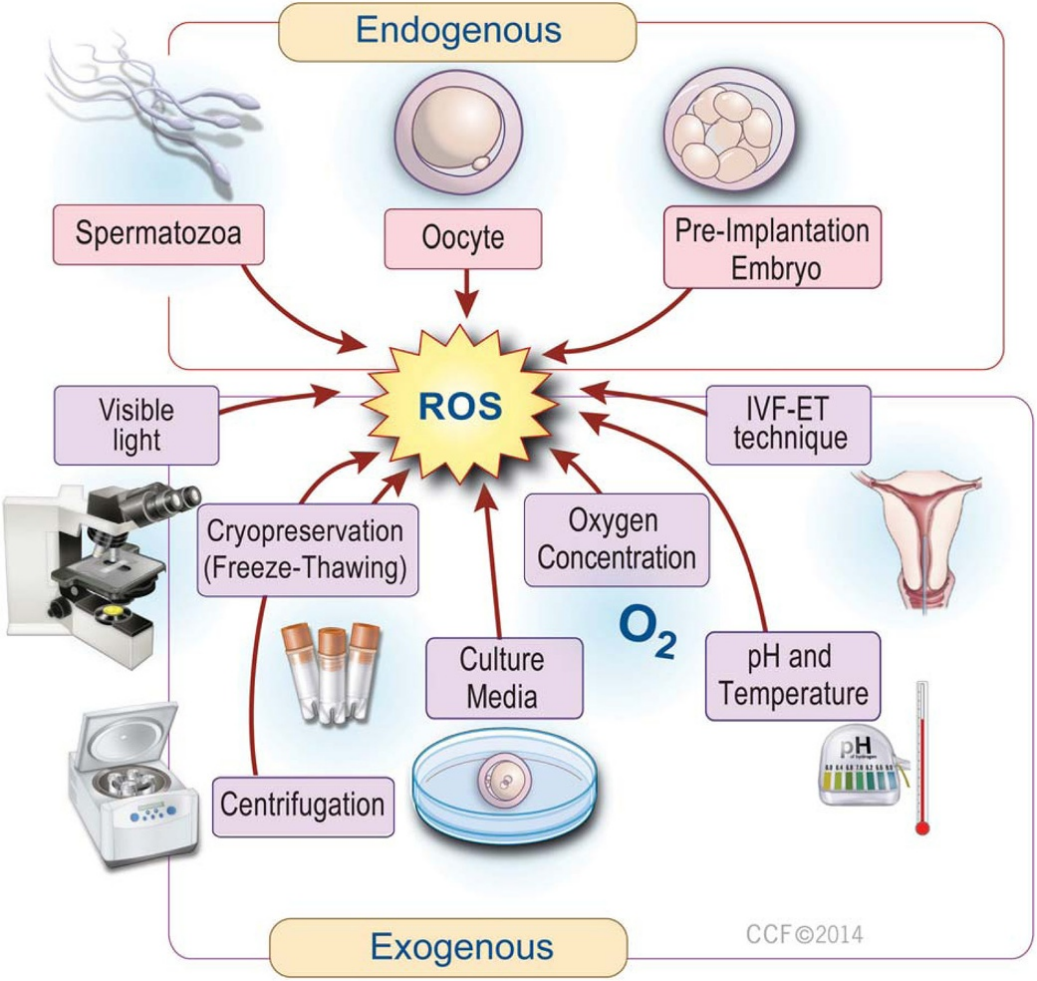
**Effects of oxidative stress (OS) and interventions to overcome its effects at different ART steps.** Overproduction of ROS could potentially occur at various steps during ART, leading to oxidative stress. Exposure of the developing embryo to oxidative stress may cause 2 cell block, reduction in blastomere numbers and blastocyst development rate, apoptosis and fragmentation of the embryo, leading to pre-implantation embryonic death. However, the appropriate intervention could be taken at various steps during ART to minimize the harmful effects of oxidative stress on ART outcome. These include: using a lower sperm concentration, shorter co-incubation periods, employment of appropriate sperm preparation techniques, the use of low levels of illumination and oxygen concentration, use of appropriate type and content of media, including supplementation with antioxidants.

Antioxidant supplementation protect spermatozoa from the effects of the freeze-thaw process [89]. For example, supplementation of cryopreservation medium with quercetin [90] and catalase [91] seemed to protect spermatozoa from oxidative stress-induced damage during the freeze-thaw process and caused improvement in spermatozoa motility, viability and DNA integrity. Addition of Vitamin E [58] and pentoxifylline [92–95] respectively to cryopreservation medium/prior to cryopreservation improved post-thaw motility. Supplementation of spermatozoa preparation medium with biotin enhanced the motility of frozen-thawed spermatozoa and prolonged its survival [96]. Post-thaw spermatozoa quality is also influenced by the cryopreservation technique and type of cryoprotectant used [97]. Figure 2 depicts the effects of oxidative stress and possible interventions to overcome its detrimental effects at different ART steps.

However, the use of newer cryopreservation techniques such as vitrification has yielded vitrified oocytes that are as viable as fresh oocytes in terms of implantation rate, embryo survival rate and clinical pregnancy rate [98]. A controlled-randomized clinical trial confirmed that vitrification is an effective form of oocyte cryo-storage and is not inferior to the use of fresh oocytes [99]. This opens a favorable avenue for patients seeking fertility preservation or those at risk of ovarian hyperstimulation syndrome.

## Antioxidants as ameliorating agents

In order to maintain physiological ROS levels and prevent oxidative stress development, excess ROS must be continuously neutralized. Antioxidants are able to neutralize pro-oxidants by either preventing its formation via termination of propagative oxidative chain reactions or by scavenging existing ROS, thereby maintaining the delicate pro-oxidant/anti-oxidant balance and consequently protecting the cell and its microenvironment from oxidative damage [4, 100]. Examples of antioxidant systems include enzymes such as SOD, catalase and glutathione peroxidase/reductase, and a variety of non-enzymes such as vitamins (E, C, B complex), polyphenols (flavonoids), carotenoids and trace minerals among others [15]. Components of the human reproductive system contain antioxidants that are either endogenously formed or acquired from dietary sources [13]. In females, antioxidants are present in the ovary, follicles, follicular, tubal and peritoneal fluid, and endometrial epithelium [6], while in males, antioxidants are found in the testis, epididymis, secretions of the male accessory organs and seminal plasma [17].

In an ART setting, antioxidants can be employed to ameliorate the harmful effects of excess ROS on gametes and embryos. Treatment strategies using antioxidants may be approached in two general modes, either as oral supplementation of the subfertile couple several months prior to their ART cycle, or as *in vitro* supplementation in media during the ART protocol itself, in order to minimize endogenous and exogenous sources of ROS, respectively. The role of antioxidants in assisted reproduction is indeed one of great importance. For example, in infertile men undergoing IVF and ICSI, high seminal ROS levels correlate negatively with spermatozoa morphology and vitality, and fertilization rates, while seminal antioxidant levels showed a positive correlation with fertilization rates. Although ROS levels were higher in IVF than in ICSI patient groups, total antioxidant concentration in seminal plasma and fertilization rates did not differ between the IVF and ICSI cohorts [9]. Seminal antioxidants, in general enhance spermatozoa quality leading to higher ART success rates. Tables 2 and 3 contain a summary of key findings of studies using oral supplementation of antioxidants respectively in men and in women.

**Table 2:** **Study outcomes involving oral supplementation of various antioxidants in men**

Antioxidant	Study type	Patient population	Intervention (daily dose x duration)	Control group (daily dose)	Study outcome (effect on sperm parameters)	Reference
*Vitamin E*	Double blind, placebo cross-over, RCT	Healthy men with high seminal ROS levels (n = 30)	600 mg vitamin E x 3 months (n = 15)	Placebo (n = 15)	Improved *in vitro* sperm function (improved zona-binding assay)	Kessopoulou et al. [101]
*Vitamin E*	Double blind, placebo-controlled	Men with (n = 110) asthenozoospermia or oligoasthenozoospermia	300 mg vitamin E x 6 months (n = 52)	Placebo (n = 55)	1. Reduced MDA concentration (less LPO in spermatozoa)	Suleiman et al. [102]
					2. Improved sperm motility	
					3. 20% of those on therapy achieved pregnancy	
*Vitamin E + Anti-Estrogen (Clomiphene citrate)*	Prospective, placebo-controlled RCT	Infertile men with idiopathic oligozoospermia (n = 60)	400 mg vitamin E +25 mg clomiphene citrate x 6 months (n = 30)	Placebo (n = 30)	1. Improved sperm count and progressive motility	Ghanem et al. [103]
					2. Partners had higher incidence of pregnancy	
*Vitamin E + selenium*	Open, randomized	Volunteers and infertile men (n = 54)	400 mg vitamin E +225 μg selenium x 3 months (n = 28)	4.5 g vitamin B x 3 months (n = 26)	1. Reduced MDA concentration (less LPO in spermatozoa)	Keskes-Ammar et al. [104]
					2. Improved sperm motility	
*Vitamin E + selenium*	Observational study	Infertile men with idiopathic asthenozoospermia (n = 690)	400 IU vitamin E +200 μg selenium x 100 days	None	1. Improvement in sperm motility/morphology or both (53%)	Moslemi & Tavanbakhsh [105]
					2. Increased spontaneous pregnancy rates (11%)	
*Vitamin E + Vitamin C*	Double blind, placebo-controlled, RCT	Men (n = 31) with asthenozoospermia or moderate oligoasthenozoospermia	1000 mg vitamin C +800 mg vitamin E x 8 weeks (n = 15)	Placebo (n = 16)	No improvement in sperm parameters	Rolf et al. [106]
					No improvement in 24 h sperm survival rate	
*Vitamin E + Vitamin C*	Observational study, double-blind	Men with elevated sperm DNA fragmentation (≥15%) who have unexplained infertility	1000 mg vitamin C +1000 mg vitamin E x 2 months (n = 32)	Placebo (n = 32)	Reduced percentage of DNA-fragmented sperm (TUNEL test)	Greco et al. [107]
*Vitamin E + Vitamin C*	Observational study involving assisted conception treatment	Men with elevated sperm DNA fragmentation (≥15%) who failed their 1^st^ ICSI attempt	1000 mg vitamin C +1000 mg vitamin E x 2 months (n = 38)	None	1. Reduced percentage of DNA-fragmented sperm (TUNEL test)	Greco et al. [108]
					2. Marked improvement in implantation and clinical pregnancy rates in the 2^nd^ ICSI attempt vs 1^st^ attempt	
*Vitamin C*		Men with sperm agglutination (>25%) (n = 30)	200 mg vitamin C or 1000 mg vitamin C	Placebo	Improved sperm motility, viability, morphology after 4 weeks (more prominent improvement in 1000 mg vitamin C vs. 200 mg vitamin C)	Dawson et al. [109]
*Vitamin C*		Men who are heavy smokers (n = 75) with normal reproductive function	200 mg vitamin C or 1000 mg vitamin C	Placebo	1. Improved sperm agglutination	Dawson et al. [110]
					2. Improved 24 h viability	
					3. Improved sperm morphology	
*Folic acid + zinc sulphate*	Double blind, placebo-controlled, RCT	Fertile (n = 108) and subfertile men (n = 103)	5 mg folic acid, 66 mg zinc sulphate or 5 mg folic acid +66 mg zinc sulphate x 26 weeks	Placebo or placebo + placebo	Increased sperm concentration in subfertile and fertile males after combined treatment	Wong et al. [111]
*Folic acid + zinc sulphate*	Double blind, placebo-controlled	Fertile (n = 47) and subfertile men (n = 40)	5 mg folic acid +66 mg zinc sulphate x 26 weeks	Placebo	Increased sperm concentration in infertile males, but not fertile males	Ebisch et al. [112]
*Folic acid + zinc sulphate*	Double blind, placebo controlled, RCT	Subfertile men with OAT (n = 83)	5 mg folic acid +220 mg zinc sulphate x16 weeks	Placebo	Zinc sulfate + folic acid did not improve sperm quality in men with OAT (severely compromised sperm parameters)	Raigani et al. [113]
*Folic acid + zinc sulphate*	Prospective, randomized controlled	Men with palpable varicocele (grade III) who underwent surgical repair of varicocele (n = 160)	5 mg folic acid (n = 26), 66 mg zinc sulphate (n = 32) or 5 mg folic acid +66 mg zinc sulphate (n = 29) x 6 months	Placebo (n = 25)	1. Zinc sulfate + folic acid improved sperm parameters and improved varicocelectomy outcomes	Azizollahi et al. [114]
					2. Improved protamine content and halo formation rate	
*Coenzyme Q*_*10*_	Systematic review and meta-analysis (3 RCTs)	Infertile men	CoQ_10_ (n = 149)	Controls (n = 147)	1. Improved seminal CoQ_10_ levels	Lafuente et al. [115]
					2. Increased sperm concentration	
					3. Increased sperm motility	
					4. No increase in pregnancy rates	
					5. Data on live births were lacking	
*Coenzyme Q*_*10*_	Double blind, placebo-controlled, RCT	Men with iOT (n = 60)	200 mg CoQ_10_ x 3 months (n = 30)	Placebo (Lactose) (n = 30)	1. Increased levels of CoQ_10_ in seminal plasma	Nadjarzadeh et al. [116]
					2. Decreased 8-isoprostane levels (biomarker of LPO) (attenuation of OS in seminal plasma)	
					3. Increased sperm forward and total motility	
					4. Increased catalase, SOD activity	
*Coenzyme Q*_*10*_	Double blind, placebo-controlled, RCT	Men with iOAT (n = 47)	200 mg CoQ_10_ x 12 weeks	Placebo	1. Reduced TBARS (reduced plasma MDA levels)	Nadjarzadeh et al. [117]
					2. Increased TAC in seminal plasma	
*Coenzyme Q*_*10*_	Double blind, placebo-controlled, RCT	Men with iOAT (n = 228)	200 mg ubiquinol x 26 weeks (n = 114)	Placebo (n = 114)	Improved sperm quality (density, motility, normal strict morphology)	Safarinejad et al. [118]
*Coenzyme Q*_*10*_	Double blind, placebo-controlled, RCT	Men with idiopathic infertility (n = 60)	200 mg CoQ_10_ x 6 months	Placebo	1. Increase in CoQ_10_ and ubiquinol in seminal plasma and spermatozoa	Balercia et al. [119]
					2. Increase in spermatozoa motility	
*Coenzyme Q*_*10*_	Prospective	Men with iOAT (n = 212)	300 mg CoQ_10_ x 26 weeks (n = 106)	Placebo (n = 106)	1. Improved sperm density, motility, normal strict morphology	Safarinejad [120]
					2. Improved acrosome reaction	
*Coenzyme Q*_*10*_	Open-label, prospective	Men with iOAT (n = 287)	600 mg CoQ_10_ x 12 months (n = 106)	None	1. Improved sperm quality (concentration, progressive motility, normal morphology)	Safarinejad [121]
					2. Improved pregnancy rates

**Table 3:** **Study outcomes involving oral supplementation of various antioxidants in women**

Antioxidant	Study type	Patient population	Intervention (daily dose x duration)	Control group (daily dose)	Study outcome	Reference
*Vitamin E*		Women with unexplained infertility undergoing ovarian stimulation and then IUI	400 IU/day vitamin E		1. Increased endometrial thickness	Cicek et al. [122]
					2. No significant increase in implantation and pregnancy rates	
*Vitamin C*	Prospective	Women undergoing IVF-ET (n = 76)	500 mg vitamin C/day (slow release) to women smokers (n = 19) and women non-smokers (n = 19)	Placebo	Women non-smokers had higher pregnancy rates than women smokers	Crha et al. [123]
*Vitamin C*	Prospective, randomized	Infertile women with luteal phase defects (not on IVF-ET)	750 mg ascorbic acid (n = 76) started on first day of third menstrual cycle until positive urine pregnancy test (maximum 6 months)	No treatment (n = 46)	1. Increase in progesterone levels	Henmi et al. [124]
					2. Increase in clinical pregnancy rates	
*Vitamin C*	Double blind, placebo-controlled, RCT	Women (<40y) undergoing first IVF-ET cycles (n = 620)	1 g or 5 g or 10 g ascorbic acid +30 mg Dydrogesteron x 14 days after follicle aspiration for IVF-ET	Placebo (Lactose + citric acid +30 mg Dydrogesteron)	No difference in clinical pregnancy and implantation rates	Griesinger et al. [125]
*Myo-inositol + folic acid*	Placebo-controlled, RCT	Infertile PCOS patients undergoing ovulation induction for ICSI (n = 60)	4 g myo-inositol +400 μg folic acid (n = 30)	400 μg folic acid only (n = 30)	Reduced germinal vesicles and degenerated oocytes without compromising the number of oocytes retrieved at ovum pick-up	Papaleo et al. [126]
*Myo-inositol + folic acid*	Double blind	Infertile PCOS patients undergoing ovulation induction for IVF or ICSI (n = 34)	4 g of myo-inositol +400 μg of folic acid, continuously for 3 months	400 μg of folic acid only	1. Greater number of oocytes recovered during pick up	Ciotta et al. [127]
					2. Greater number of oocytes with score S1	
					3. Reduced number of immature oocytes (vesicles germ and degenerated oocytes)	
*Melatonin*	Follicular fluid sampled during oocyte retrieval during IVF-ET	Women with prior failure of IVF-ET cycle and who are attempting IVF-ET again (n = 115)	3 mg melatonin (n = 56) given on the 5th day of the previous menstrual cycle until the day of oocyte retrieval	Without melatonin treatment (n = 59)	1. Improved fertilization rate compared to previous IVF-ET cycle	Tamura et al. [128]
					2. Improved oocyte quality	
*Melatonin*	Prospective, randomized	Women with primary infertility undergoing IVF-ET cycles (n = 85)	3 mg melatonin (n = 40) administered continuously from day of GnRH	No treatment (n = 45)	1. Higher percentage of morphologically mature oocytes retrieved (MII oocytes)	Batioglu et al. [129]
					2. Higher mean number of top quality (class I) embryos	
					3. No improvement in fertilization rates	
					4. Higher tendency of clinical pregnancy rate (not statistically significant)	
*Melatonin*	Prospective, randomized	IVF patients with disturbed sleep (insomnia) who were undergoing IVF-ET (n = 60)	3 g melatonin (n = 30) given 3rd to the 5th day of the previous menstrual cycle until the hCG injection day of the controlled ovarian hyperstimulation	No treatment (n = 30)	1. Higher mean number of the retrieved oocytes, mean MII oocyte counts, and G1 embryo ratio	Eryilmaz et al. [130]
					2. No change in sleeping status	
*Melatonin + myo-inositol + folic acid*	Prospective, randomized	Women undergoing IVF cycles (n = 65)	3 g melatonin +4 g myo-inositol +200 mg folic acid (n = 32) administered continuously from day of GnRH	4 g myo-inositol + folic acid (n = 33)	1. Greater mean number of mature oocytes (and lower mean number of immature oocytes)	Rizzo et al. [131]
					2. Higher mean number of top quality embryos (class 1 and 2)	
					3. No improvement in fertilization rates	
					4. Higher tendency of clinical pregnancy rate and implantation rate (not statistically significant)	
*Melatonin + myo-inositol + folic acid*	Prospective, longitudinal, cohort	Women with failed IVF cycle (due to poor oocyte quality) who were undergoing a new IVF cycle	3 mg melatonin +4 g myo-inositol +400 mcg folic acid x 3 months (n = 46)	Prior cycle of the same women but without treatment	1. Higher number of morphologically mature oocytes retrieved (MII oocytes)	Unfer et al. [132]
					2. Higher total number of embryo transferred and higher number of top quality (score 1 & 2) embryo transferred	
					3. Increased fertilization rate	

### Enzymatic antioxidants

In normozoospermic men, higher seminal activities of enzymatic antioxidants correlate with lower total malondialdehyde levels (MDA), indicating the protective effect of these antioxidants against oxidative stress-induced lipid peroxidation [133, 134]. Conversely, infertile men with poor spermatozoa quality have lower levels of seminal enzymatic antioxidants that correspond with increased levels of lipid peroxidation [133, 135].

#### Superoxide dismutase

Isoforms of SOD found in mitochondria (manganese SOD) and cytoplasm (copper/zinc SOD) provide fundamental defense against ROS [136]. Normozoospermic men have higher seminal SOD activity compared to men with abnormal sperm parameters. Seminal SOD activity correlates positively with spermatozoa concentration and motility and inversely with both sperm DNA fragmentation and semen volume [137, 138]. SOD activity is present in granulosa and theca interna cells of pre-antral, antral and in dominant follicles, with increasing expression towards ovulation [139]. Women with tubal factor infertility who failed to conceive had lower SOD activity in their granulosa cells and a reduction in embryo quality [140]. Furthermore it was shown that higher SOD activity in cumulus cells from women with infertile partners lead to better ART outcome. Thus, SOD activity may be indicative of a better quality oocyte during oocyte selection [31].

#### Catalase

Found in cellular peroxisomes, catalase decomposes hydrogen peroxide to oxygen and water [141]. Catalase, originating mainly from the prostate gland, is present in seminal fluid and motile spermatozoa [142]. Low seminal catalase activity poses a greater risk of post-fertilization failure in infertile couples undergoing IVF [143]. Catalase added to spermatozoa preparation media resulted in reduced ROS and DNA fragmentation levels and an increased acrosome reaction rate in spermatozoa from normozoospermic men [144]. During spermatozoa cryopreservation, supplementation of cryomedia with catalase gave better freeze-thaw outcomes, with higher spermatozoa motility, vitality and lesser DNA damage [91, 145]. Similarly, co-supplementation of cryopreserved spermatozoa with catalase and SOD gave higher post-thaw motility and viability [146].

#### Glutathione system

The glutathione enzymatic family comprises reduced glutathione (GSH), glutathione peroxidase (GPx, isoforms Gpx 1 to GPx 6), glutathione-S transferase (GST) and glutathione reductase (GR). GSH is a non-enzymatic antioxidant peptide formed in the cytosol from glycine, cysteine, and glutamate. GSH is oxidized to gluthathione disulphide (GSSG) by GPx. There are 6 isoforms of GPx: the selenocysteine-containing GPx1 to GPx4, and selenium-independent GPx5, which is present in the epididymis. All GPx isozymes reduce hydrogen peroxide and lipid hydroxyperoxides [147, 148]. Seminal GPx activity is lower in infertile men with abnormal spermatozoa quality, but no correlation was found between GPx levels and spermatozoa fertilization potential or pregnancy rates in IVF [149].

### Non-enzymatic antioxidants

#### Vitamins and vitamin-like substances

##### Vitamin E

Vitamin E is a naturally-occurring, lipid-soluble antioxidant. It’s most active form, alpha-tocopherol quenches hydrogen peroxide, superoxide anion, hydroxyl anions and breaks peroxidation chain reactions. RCTs and prospective studies concur that oral supplementation of vitamin E reduces lipid peroxidation damage [102, 150], improves sperm motility [102, 103] and function [101] as well as improve fertilization [150] and pregnancy rates [102, 103]. In a small prospective study (n = 15), Geva’s group showed that oral vitamin E (200 mg for 3 months) increased oocyte fertilization rate per IVF cycle in fertile, normozoospermic men who initially had low fertilization rates during a previous IVF attempt. Following antioxidant therapy with vitamin E, these men also experienced lower lipid peroxidation levels in their spermatozoa [150]. In women with unexplained infertility, oral vitamin E intake improved the endometrial response, possibly due to its antioxidant and anticoagulant effects as well as by modulating the anti-estrogenic effect of clomiphene citrate. However, no differences in implantation or pregnancy rates were observed [122]. The *in vitro* effects of vitamin E on normal and abnormal spermatozoa during cryopreservation are improved post-thaw motility [57, 58] and DNA integrity [57], while addition during incubation improved motility and viability of abnormal spermatozoa [59].

Selenium, an essential micronutrient and a free radical-scavenger, works synergistically with Vitamin E to protect spermatozoa from the effects of oxidation [104] and to improve motility [104, 105], morphology and pregnancy rates [105]. Found in high concentrations in testicular tissue, selenium is required for testosterone synthesis and spermatogenesis [151, 152]. Moslemi and Tavanbakhsh studied the effects of vitamin E and selenium therapy for 14 weeks in 690 asthenoteratozoospermic infertile men from couples with male factor infertility. Semen analysis was found to be improved in 362 or 52.6% patients: 299 patients showed improved motility, 21 patients showed improved morphology and 42 patients showed improvement in both sperm motility and morphology. However, 253 cases (36.6%) showed no change in their semen analysis, while the remaining 75 patients (10.8%) achieved spontaneous pregnancy [105]. Based on their findings, it seems that a combination of vitamin E and selenium had a more significant impact on motility compared to morphology.

Besides selenium, vitamin E is often administered in combination with vitamin C, another chain breaking antioxidant. An initial RCT reported that sperm parameters did not change after co-supplementation with vitamins E and C [106]. However, subsequent observational studies in men with poor sperm DNA integrity showed that supplementation with both vitamins E and C resulted in fewer spermatozoa with fragmented DNA [107, 108], as well as higher implantation and clinical pregnancy rates [108].

##### Vitamin C

Vitamin C (L-ascorbic acid, ascorbate) is a water-soluble, naturally-occurring, chain-breaking antioxidant. It is unstable, easily oxidized and perishable in high temperatures [153]. Ascorbic acid taken as dietary intake [154, 155] or oral therapy, improves spermatozoa quality [109, 110]. In a large, placebo-controlled, double blind RCT, vitamin C supplementation for a period of 14 days starting on the day of follicle aspiration in women undergoing IVF-ET showed no improvement in clinical pregnancy or implantation rates [125]. However, smaller prospective studies showed that oral vitamin C supplementation in women, either undergoing IVF-ET treatment [123] or with luteal phase defects [124], lead to increased pregnancy rates. Addition of vitamin C in cryomedia improved motility [62] and reduced DNA damage [61] in post-thaw spermatozoa. Similarly, vitamin C supplemented culture media reduced lipid peroxidation and DNA damage, while improving spermatozoa motility and viability [60].

##### Vitamin B – folic acid

B vitamins form a group of water soluble antioxidants. Folate (vitamin B9) is the natural dietary form, while folic acid is its synthetic equivalent. Folate levels in seminal plasma are higher than in serum and in fertile men compared to infertile men [156, 157]. Similarly, fertile men were found to have higher seminal zinc levels compared to infertile men [158]. As it has been observed that zinc deficiency decreases the absorption and metabolism of folate, most studies related to folate is combined with zinc supplementation. An essential trace element, zinc acts as a ROS-scavenger and regulates sperm motility [159]. In men with asthenozoospermia, intake of zinc sulphate (500 mg for 3 months) improved spermatozoa quality (count, progressive motility and fertilizing capacity) and reduced the incidence of antisperm antibodies [160].

Double-blind, placebo-controlled, RCTs investigating the effects of combined folic acid and zinc sulphate oral treatment in infertile men report an increase in sperm concentration [111, 112], but not in those with severe oligoasthenoteratozoospermia (OAT) [113]. Among varicocele patients who sought surgical intervention, combined therapy of folic acid and zinc sulphate improved sperm parameters and consequently varicocelectomy outcome [114]. In infertile women with PCOS undergoing ovulation induction for IVF/ICSI, oral therapy of myo-inositol (a component of the vitamin B family) and folic acid reduced the number of immature oocytes during pick up [126, 127].

##### Coenzyme Q_10_

Coenzyme Q_10_ is a vitamin-like, lipid soluble substance present in most eukaryotic cells as it forms part of the the mitochondrial respiratory chain. Coenzyme Q_10_ may also be present in its oxidized (ubiquinone) or reduced form (ubiquinol) [161]. Results of a systematic review and meta-analysis on coenzyme Q_10_ therapy in male infertility show that oral supplementation with coenzyme Q_10_ increased seminal coenzyme Q_10_ levels, spermatozoa concentration and motility. However, there was no increase in pregnancy rates while data for live births was lacking [115]. In 4 double-blind, placebo-controlled RCTs using coenzyme Q_10_ or ubiquinol therapy in men with idiopathic infertility, study outcomes also reported of lower lipid peroxidation and oxidative stress levels in seminal plasma [116, 117], increase in seminal enzymatic antioxidant activity [116, 117] and ubiquinol (a potent antioxidant) levels [119]. Prospective studies on coenzyme Q_10_ intake in men with idiopathic infertility reported of improved acrosome reaction [120] and pregnancy rates [121]. In infertile men with prior failed IVF/ICSI, coenzyme Q_10_ supplementation increased fertilization rates in the subsequent cycle [63]. The group also found that adding coenzyme Q_10_ into media with asthenozoopermic spermatozoa increased spermatozoa motility [63].

#### Hormones

##### Melatonin

Melatonin, a powerful antioxidant secreted by the pineal gland, is present in follicular fluid and semen. Melatonin also activates the primary enzymatic antioxidants (SOD, catalase, GPx) [162]. Interestingly the concentration of melatonin in the pre-ovulatory follicle is higher than normal plasma melatonin levels [163]. Intrafollicular melatonin levels also inversely correlated to 8-OHdG and thus degenerate oocytes [164]. In several prospective randomized studies in infertile women undergoing IVF-ET cycles, continuous oral melatonin supplementation, starting from the previous menstrual cycle until ovarian stimulation, improved oocyte quality, increased the number of mature MII oocytes retrieved and resulted in a better ratio of top quality embryos [129, 130, 164]. In other prospective studies, melatonin was given along with myo-inositol and folic acid in women planning for IVF treatment, which also resulted in higher number of mature oocytes and top quality embryos [131, 132]. There was a tendency for higher clinical pregnancy and implantation rates (although statistically insignificant) [129, 131], while fertilization rates were reported to be either higher [132, 164] or without improvement in [129, 131]. However, a systematic review and meta-analysis of RCTs on melatonin supplementation in women undergoing controlled ovarian stimulation for assisted conception concluded that the included trials provided low quality evidence on the parameters examined [165]. In experimental *in vitro* studies, spermatozoa from healthy men incubated with melatonin showed improved motility, viability [64, 65] and higher ratio of spermatozoa with normal morphology [64].

#### Other antioxidative substances

Trace element supplementation has shown to improve sperm quality. For example, *in vitro* zinc supplementation to sperm media, either alone or in combination with other antioxidants (as previously discussed), reduced sperm DNA fragmentation [166, 167], and loss of motility [167]. Addition of zinc to cryomedia also protects against post-thaw loss of spermatozoa function and sperm DNA damage [168]. In a large double blind placebo-controlled RCT involving infertile men with idiopathic OAT (iOAT) combined oral intake of selenium and N-acetyl-cysteine correlated positively with spermatozoa quality. The additive effects were significantly better when compared to either selenium or N-acetyl-cysteine intake [169]. Besides trace elements, other antioxidants such as L-Carnitine also play a role in the enhancement of sperm parameters. Derived from lysine, L-Carnitine is a naturally-occurring molecule [170] that scavenges ROS. A systemic review and meta-analysis of 9 RCTs concluded that oral supplementation with L-carnitine or L-acetyl-carnitine improves total sperm motility and pregnancy rates [171]. In two separate studies, it was shown that the *in vitro* addition of L-carnitine to the culture media not only improved oocyte chromosomal structure and reduced embryo apoptosis [66], but also improved blastocyst development rate [67].

## Conclusions

A Cochrane review on antioxidant intake in male partners of couples undergoing ART (34 trials, 2876 couples) reported increased pregnancy rate (15 trials, 964 couples, 96 pregnancies) (pooled odds ratio (OR) 4.18, 95% CI 2.65-6.59; P < 0.00001, I^2^ = 0%) and increased live births (3 studies, 214 couples, 20 live births) (pooled OR 4.85, 95% CI 1.92-12.24); P = 0.0008, I^2^ = 0%) in men taking oral antioxidants [172]. On the other hand, a Cochrane review on oral antioxidant supplementation in women seeking IVF/ICSI (28 trials, 3548 women) reported of very low quality evidence indicating that antioxidant intake was neither associated with increased pregnancy rate (13 trials, 2441 women) (OR 1.30, 95% CI 0.92-1.85; P = 0.14, I^2^ = 55%) nor with live birth rate (2 trials, 97 women) (OR 1.25, 95% CI 0.19-8.26; P = 0.82, I^2^ = 75%). However, data from 3 trials (276 women) showed that pentoxifylline was associated with increased clinical pregnancy rates (OR 2.03, 95% CI 1.19-3.44, P = 0.009, I^2^ = 0%) in subfertile women [173]. Both these reviews outlined the need for better quality evidence that would allow for a more definitive verdict on the usefulness of oral antioxidant therapy in the ART population.

Along these lines, further large, well-designed randomized controlled clinical trials on oral supplementation of antioxidants is required in order to give stronger evidence and determine more conclusively regarding the safety and efficacy of antioxidant therapy in improving gamete quality in infertile males and females as well as couples seeking ART. Similarly, the use of antioxidants *in vitro* in the clinical laboratory setting during ART procedures should also be considered, alongside improvement of ART techniques and optimization of the laboratory environment. As even some of the studies that form the basis for the previously listed Cochrane reviews are subject to significant heterogeneity it is important to note that unrestricted recommendation of antioxidant supplements could even be hazardous to patients.

Undeniably, excessive ROS leading to oxidative stress conditions has a serious impact on the outcome of assisted reproduction, leading to lower fertilization, implantation and pregnancy rates. As highlighted in this review, ART procedures *in vitro* presents with many avenues for ROS and oxidative stress development, which would negatively impair gamete/embryo quality and consequently reduce ART success. While the generation of ROS during ART steps cannot completely be avoided, practical strategies that minimize potential ROS-inducing factors during the ART procedures, as portrayed in this paper, are worth exploring.

In conclusion, prophylactic oral antioxidant therapy and supplementation of medium for culture, incubation/handling and cryopreservation can possibly help improve gamete quality and fortify the developing embryo. However, the appropriate antioxidants and dosages (whether as a sole compound or as a combination) suitable for different forms of infertility issues still remain an ongoing area of research.

## Abbreviations

ART: Assisted reproductive technology
CoQ_10_: Coenzyme Q_10_
GnRH: Gonadotropin releasing hormone
GSH: Reduced Glutathione
GPx: Glutathione peroxidase
GR: Glutathione reductase
GST: Glutathione-S transferase
GSSG: Gluthathione disulphide
hCG: Human chorionic gonadotropin
IUI: Intrauterine insemination
IVF: *In vitro* fertilization
IVF-ET: *In vitro* fertilization-embryo transfer
ICSI: Intracytoplasmic sperm injection
LPO: Lipid peroxidation
OAT: Oligoasthenoteratozoospermia
OS: Oxidative stress
PCOS: Polycystic ovarian syndrome
RCTs: Randomized controlled trials
ROS: Reactive oxygen species
SOD: Superoxide dismutase.
